# Retraction: Ftx non coding RNA-derived miR-545 promotes cell proliferation by targeting RIG-I in hepatocellular carcinoma

**DOI:** 10.18632/oncotarget.28695

**Published:** 2025-02-18

**Authors:** Zhikui Liu, Changwei Dou, Bowen Yao, Meng Xu, Linglong Ding, Yufeng Wang, Yuli Jia, Qing Li, Hongyong Zhang, Kangsheng Tu, Tao Song, Qingguang Liu

**Affiliations:** ^1^Department of Hepatobiliary Surgery, The First Affiliated Hospital of Xi’an Jiaotong University, Xi’an, Shaanxi, China; ^*^These authors have contributed equally to this work

**This article has been retracted:** Oncotarget has completed an investigation of this paper and found that several images were duplicated and reused in other papers originating from the same lab. Specifically, Figure 9A contains a duplicate image of mice used in Figure 7A of their earlier paper [1]. In addition, Figure 8, panels A, B and G, contains several duplicate GAPDH loading control bands from Figure 7, panels A and B of another paper published concurrently [2]. Also, the IHC image from this paper (Figure 6G) was reused to illustrate a different experiment in the later published paper from the same lab [3]. Furthermore, the National Natural Science Foundation of China Supervision Committee has launched an investigation into the suspected academic misconduct of different papers published by Liu Zhikui, Liu Qingguang, Tu Kangsheng, Yang Nan and others from Xi’an Jiaotong University and it was found that this particular paper had problems such as plagiarism, improper manipulation of images, and confusion in the use of images. The investigation demonstrated that the first author, Liu Zhikui, and the corresponding authors were responsible for the problems in the paper. The National Natural Science Foundation of China applied different administrative actions to the above-mentioned authors based on the results of the investigation of their papers published in different journals. In light of these facts, the Editorial decision was made to retract this paper. All authors have agreed to the decision.

Original article: Oncotarget. 2016; 7:25350–25365. 25350-25365. https://doi.org/10.18632/oncotarget.8129
